# How to prevent equity efforts from losing steam in global health academia

**DOI:** 10.1371/journal.pgph.0001656

**Published:** 2023-03-08

**Authors:** Shashika Bandara, Ananya Tina Banerjee

**Affiliations:** 1 Faculty of Medicine and Health Sciences, Department of Family Medicine, McGill University, Montreal, Canada; 2 Faculty of Medicine and Health Sciences, School of Population and Global Health, McGill University, Montreal, Canada; PLOS: Public Library of Science, UNITED STATES

In the wake of the killing of George Floyd on May 25, 2020, there was a strong call to action, first in North America and then globally, to tackle racism and promote racial justice [1]. Many universities created “equity, diversity, inclusion, anti-racism (EDI-AR)” task forces and other mechanisms with goals of transforming institutions entrenched in histories of colonialism and white supremacy. Concurrently, decolonizing global health movements have been gaining momentum asking all stakeholders including academic institutes to advance equity and dismantle systemic forms of oppression [2,3].

Often equity efforts initiated during watershed moments face the challenge of losing team. The crescendos and unavoidable reckoning with every individual’s relationship to structural injustices ultimately dwindle and the status quo in academia continues to instill ingrained systems of discrimination [4]. Deliberate equity building in academia including decolonizing curricula and institutional practices are also highly relevant to addressing structural fault lines in global health governing models, implementation practices and research partnerships [5]. Lessons from failures in Covid-19 pandemic and climate crisis highlight the urgency to better equip students and faculty to lead equity centered approaches [6].

In this essay, we highlight six key considerations that senior leadership and community at large within high income country (HIC) global health academic institutions need to be mindful to successfully embed equity efforts into the fabric of their foundation. While our experiences as authors stem largely from our engagement in global health academia, the following reflections cut across disciplines.

## Listening is necessary but not sufficient

Many universities and organizations have now collected information on aspects the institute needs to improve upon [7]. However, often after this stage of ‘information collection’ many equity efforts remain in administrative limbo citing either lack of work force or resources for institutional change. This can be harmful and traumatic to those who chose to provide often unpaid labor to examine ways forward to change a colonialist system. Additionally, it is important that, intentionally or unintentionally, we do not recreate discriminatory practices of administrative delays, resulting in nothing more than mere tokenism, falling short of delivering any real value. Therefore, equity efforts start with listening *and* acting on what has been said with a sense of urgency, awareness, and commitment to bridge public and global health divides.

## Avoid the ‘defensive crouch’ when facing criticism

Many EDI-AR and decolonizing efforts are built as a response to criticism of an institute’s discriminatory practices and colonial legacies. These could be lack of diversity hires, exclusion of colonial, Black, Indigenous history in core curriculum, undermining equity in the research agenda, or dismissing inclusive community engagement that impact diverse student groups or faculty and so on. When challenges are confronted, it is crucial not to be defensive and promote resistance on the need for power, control, or concealment. Understanding that equity efforts are ever-evolving and require ongoing investment is paramount for success. Recognizing the privileges we hold that can blind us to the systemic challenges that others face is a first step in advancing equity. This privilege is not limited to dominant social groups. Many within marginalized social groups also benefit from classism, legal citizenry, family wealth and so on [8,9]. Therefore, avoiding ‘the defensive crouch’ both personally, professionally and at an institutional level can help advance collective equity efforts rapidly.

## Equity building: More than a collage of diverse faces

One of the popular methods that organizations and teams use to showcase their diversity is to include people of color in photos, websites, and videos. Ethos of equity are not automatically included by having a token group of diverse people [10]. In fact, the opposite could occur where team members from diverse origins can perpetuate inequitable practices due to lack of awareness, introspection, and training. Therefore, academic institutions and teams within should invest in training and intentionally integrating equity building steps in their internal practices as well as their outward facing practices (e.g., practices with partners). Some of these practices can include making an effort to understand challenges diverse team members face by learning from each other (e.g., immigration challenges, diverse systems of oppression), encouraging team members to recognize their privileges and improving their contribution to advance equity within the organization. These practices should also include consistent hiring of faculty dedicated to equity building, especially persons of color with lived experiences informing inclusive research, teaching, and institutional practices.

## Care for those who care for your community

Equity building efforts require significant investment of time both at professional and personal capacities and often fall on those who are systemically marginalized or allies who are faced with being the ‘first only different’ person [11]. The negative outcomes of not supporting Chairs, Leads, members on committees involved in equity building efforts can range from burnout to depression. This is more prevalent in Indigenous and racialized women who continue to be undervalued despite the increased focus on racial equity [12]. To address these challenges first there needs to be a culture of shared responsibility to raise the profile of equity building as a priority agenda item within *all* activities of academic institutes. One example of a best practice is Canadian Society of Immunology opening its 2021 conference with EDI training with a goal of seeding equity centered behavior in the community [13]. The outcome of such efforts will help hardwire equity building as a core aspect that students, faculty and staff engage with—than considering it as an afterthought. Additionally, there needs to be institutional allyship through supportive personal relationships, acts of sponsorship and collective advocacy for those who lead equity efforts.

## Put money behind pledges

We cannot replicate the current global health organizational models built on highly paid white leadership and inexpensive marginalized labor that result in adverse work conditions [14]. Governing bodies and funders need to recognize the importance of fully investing in equity. This investment needs to include an EDI-AR Office to advance unit-specific goals in addressing inequities and serving to support diverse faculty, students, and staff (as opposed to one paid position or a budget for educational events and optional trainings). The intent of a HIC academic institute is best illustrated by the financial investments they make towards the goals they want to achieve. EDI-AR pledges without meaningful resources are destined to fail. Given that the challenges threatening the health of the globe and humanity require equity building from academia to global structures, we argue that there is a strong business case for investing more in equity building from a pragmatic perspective, if nothing else [15].

## Communicate your wins, your losses, and your timelines

Communicating your progress to the wider community and making transparency a central part of your equity building efforts is beneficial in multiple ways. First, it provides confidence in those who are often affected by lack of equity that there is progress being made, despite challenges. Second, it also inspires those who are interested in supporting these efforts creating an environment of shared responsibility and influx of energy. Third, understanding that success is a non-linear process, providing a tentative plan will help hold institutions accountable while recognizing opportunities to improve. We are building from a place of imperfection; communicating plans, progress of EDI-AR and decolonizing efforts should not be held back out of fear or with the intent of achieving perfection.

## Conclusion

Equity is a continuous process, and we must all shift our conceptions of senior leadership beyond the hierarchal notions toward more relational and collectivist ways that recognize shared responsibility. We must actively work to transform colonial structures and normative practices that perpetuate injustices within academic institutes.
